# Health profiles of 996 melanoma survivors: the M. D. Anderson experience

**DOI:** 10.1186/1471-2407-6-95

**Published:** 2006-04-18

**Authors:** Charles Stava, Martha Beck, L Todd Weiss, Adriana Lopez, Rena Vassilopoulou-Sellin

**Affiliations:** 1 Department of Endocrine Neoplasia and Hormonal Disorders, Unit 435, The University of Texas M. D. Anderson Cancer Center, 1515 Holcombe Blvd., Houston, TX 77030, USA; 2 Department of Biostatistics and Applied Mathematics, Unit 447, The University of Texas M. D. Anderson Cancer Center, 1515 Holcombe Blvd., Houston, TX 77030, USA

## Abstract

**Background:**

The incidence and survival of melanoma are increasing, but little is known about its long-term health effects in adult survivors.

**Methods:**

A health survey was available from 996 melanoma survivors (577 treated with surgery alone, and 391 with combined treatments). Their medical/physiologic and psychosocial responses were analyzed and compared with those of the survivors from other cancers.

**Results:**

The melanoma survivors were 44.8 ± 12.8 years of age at diagnosis (significantly younger than the survivors of other cancers) and 63.7 ± 12.8 years at survey. Melanoma survivors were less likely to report that cancer had affected their health than survivors of other cancers (15.8% vs. 34.9%). The 577 individuals treated with surgery alone reported arthritis/osteoporosis, cataracts, and heart problems most frequently (less often than survivors of other cancers). The 391 individuals who had undergone combined treatments reported circulation problems and kidney problems generally as often as survivors of other cancers. Health problems were not associated with number of decades since diagnosis but with age at diagnosis, treatment modality, and family relationships.

**Conclusion:**

We present information from a large cohort of long-term survivors of melanoma. As a group, they were less likely to report that cancer had affected their overall health than survivors of other cancers; a number of disease related and psychosocial factors appear to influence their health profiles.

## Background

The proportion of the population who consider themselves cancer survivors has grown in recent years and will continue to increase, partly because of improvements in early detection, treatment, and supportive care. The National Cancer Institute's Office of Cancer Survivorship reported an estimated 9.8 million cancer survivors in 2001, representing 3.5% of the nation's population [1]. At least 14% of these cancer survivors were diagnosed more than 20 years ago. Indeed, there is a cancer survivor in one of every four families in the United States. As more people survive cancer, more attention is being given to understanding the long-term effects of cancer and cancer therapy on the health of cancer survivors. However, research on cancer survivorship in adults has been generally limited to conventional treatment options and their side effects rather than the long-term outcome of survivors.

Of particular interest to us was the effect of melanoma and its treatment on survivors, as melanoma is the sixth most common type of cancer in American men and the seventh most common in American women [2]. The American Cancer Society estimated that about 59,580 new cases of melanoma would be diagnosed in the United States during 2005 [3]. The estimated lifetime risk in the United States is one case in 75 people, [4] with a mean increase of 3% in new diagnoses per year [5,6]. At the same time, the survival of patients with melanoma has improved significantly; although 5-year and long-term survival rates vary by disease stage at the time of diagnosis, the overall 5-year survival for melanoma has risen to 89% [7]. Likely contributing factors to this improvement are better education, earlier detection, and more successful combination treatment regimens [8]. Therefore, the number of individuals previously diagnosed and treated for melanoma is expected to rise significantly during the coming years. Accordingly, it becomes increasingly important to define what, if any, medical and psychosocial problems characterize this particular group of cancer survivors. Indeed, a major publication recently released by the Institute of Medicine recognized the great need for systematic and comprehensive follow-up care and analysis of cancer survivors [9].

To address the growing need for objective data analysis of cancer survivors for their long-term sequelae, we initiated the Life After Cancer Care at The University of Texas M. D. Anderson Cancer Center. In the present report, we have utilized information gathered from this program to describe the long-term health profiles of melanoma survivors.

## Methods

Our data were derived from a health survey that had been developed to collect information on the long-term health effects of cancer and its treatment among cancer survivors, the detailed methods of which have been published previously [10]. In brief, an intake assessment tool used by patients of the outpatient clinics at M. D. Anderson Cancer Center provided the blueprint for the survey. Surveys had been available online and had been mailed to former M. D. Anderson patients who had been known to have been alive more than 5 years after diagnosis and who no longer required active treatment (as identified through our institution's Department of Medical Informatics database). Only individuals age 18 years or older were included. We were especially interested in looking at patients that were treated at M.D. Anderson, and because we had previously noted significant differences between the results of online and mailed surveys [10], we also excluded surveys sent via our online form. In addition, individual medical record review would only be possible for former M.D. Anderson patients. The surveys had been mailed to the survivors' last known addresses with a self-addressed stamped envelope enclosed for their return. Data were collected for 3 years. The institutional review board at M. D. Anderson had approved this survey.

Respondents were asked to provide demographic data, including marital status, ethnicity, education, employment, health care access, age at diagnosis, and age at the time of the survey, and information on family and intimate relationships. They were also asked if they believed cancer had affected their overall health and were given the opportunity to specify from a checklist the specific health conditions or symptoms they had experienced since their cancer treatment. The checklist specified abdominal pain, arthritis/osteoporosis, bleeding, cataracts, circulation problems, diabetes/sugar in the urine, dizziness, frequent infections, hearing loss, heart problems, HIV/AIDS, kidney problems, liver problems, loss of memory, lung problems, migraine headaches, psychological/psychiatric problems, second primary cancers, seizures, skin disorders, stroke, and thyroid problems. As mentioned earlier, the checklist of health conditions were gleaned from an intake assessment distributed to patients at M.D. Anderson's outpatient clinics. The survey also had specific questions about psychosocial elements such as family relationships and employment.

The long-term health effects of cancer and its treatment on survivors of melanoma and other cancers were compared. In an effort to tease out the effects of cancer treatment versus effect of age in the health problems that the survivors reported, we also compared the responses of melanoma survivors who only had surgery versus those who also received chemotherapy and/or radiotherapy (combined treatments). We might expect that health problems reported by survivors of surgery alone would tend to reflect age more than treatment effects. We also conducted direct medical record review of melanoma survivors who reported that they had received combined treatments.

Data collected from the survey are stored in an Oracle Enterprise Edition database (version 8.1.5; Oracle, Redwood Shores, CA), which is maintained and updated on a regular basis by M. D. Anderson's information technology team and the Life After Cancer Center team. Statistical analyses were performed with Statistica for Windows (version 6.1; StatSoft, Tulsa, OK). Frequency tabulations and histograms were used to fully describe the distribution of responses. Descriptive statistics, including frequency, percentage, mean, standard deviations, median and range, were used to summarize the survey data. Multivariate logistic regression methods were used to simultaneously evaluate the effects of cancer type (melanoma vs. non-melanoma), and treatment (surgery only vs. combined treatment) on the probability a patient believed that cancer had affected overall health (health affected vs. not health affected) while controlling for age at diagnosis, time from diagnosis, gender, marital status and ethnicity. All statistical tests were two-sided at a significance level of 5%. All analyses were performed using SAS Version 9.1 for Windows (SAS Institute, Cary, NC) and Statistica Version 6.1 for Windows (Tulsa, OK).

## Results

### Population

**Figure 1:**
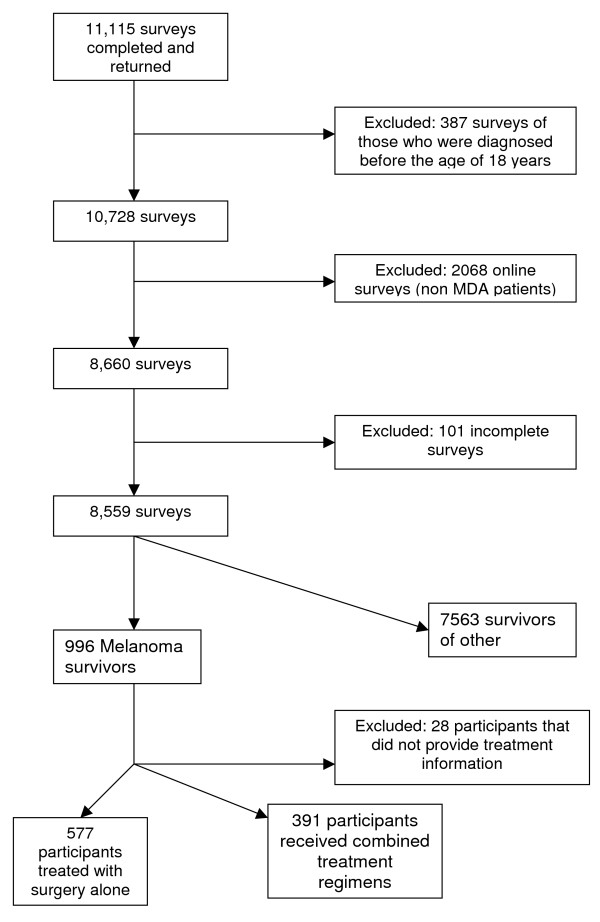
Filtering of Study Population.

After three years, 11,115 surveys had been completed and returned. We used the data collected in these surveys. Because our focus was adult survivors of cancer, we excluded the 387 surveys from survivors who had been diagnosed with cancer before the age of 18 years. We excluded 2068 surveys sent via our online form. Also excluded were 101 surveys with incomplete data or that did not meet all the inclusion criteria. As a result, we narrowed the population to 8559 cancer survivors; of these, 996 (11.6%) had been previously diagnosed with melanoma. The remaining 7563 were survivors of other cancers. Of the 996 participants, 577 had undergone surgery alone, and 391 had undergone combined treatment. Twenty-eight individuals had not provided treatment information and were excluded from the treatment analysis. Figure 1 features the flowchart delineating the filtering process. In addition, we compared the melanoma survivors to the general population represented in a recent national health survey.

**Figure 2:**
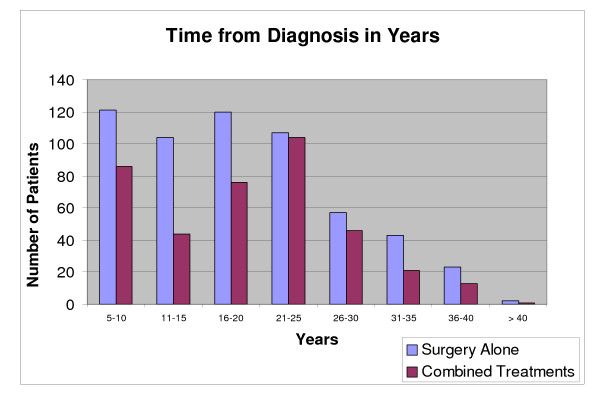
Time from Diagnosis in Years.

**Figure 3:**
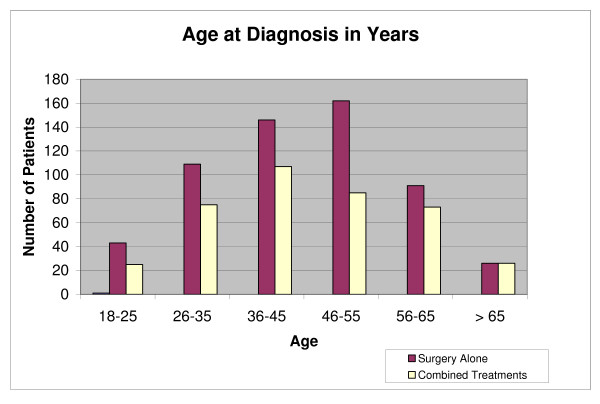
Age at Diagnosis in Years.

The overall sample of melanoma survivors included 530 women (53.2%) and 466 men (46.8%) compared with 61.8% and 38.2%, respectively, for survivors of other cancers. Most (97.9%) identified themselves as white Americans, which reflects M. D. Anderson's patient population. The mean age at the time of diagnosis was 44.8 ± 12.8 years, which was younger than the mean age for the survivors of all other cancers (49.4 ± 14.2 years) (Table 1). The time from diagnosis to survey was 18.8 ± 8.8 years, which was significantly longer than the interval since diagnosis for survivors of all other cancers (15.2 ± 8.9 years). Figure 2 shows the distribution of time from diagnosis in years for the two treatment groups. There was no significant difference by sex (data not shown). The mean age at the time of the survey for the melanoma survivors was 63.7 ± 12.8 years. The men were older at the time of diagnosis than the women (mean age, 47.1 ± 12.6 vs. 42.8 ± 12.6 years, respectively). Figure 3 shows the distribution of ages at diagnosis for the two treatment groups. Melanoma had been diagnosed at least 5 years earlier for all (by design) and more than 25 years earlier in 26.6% of patients who had filled out the survey, providing information about long-term effects.

### Effects of cancer on overall health

After adjusting for cancer treatment, years from diagnosis, and socio-demographic factors such as age at diagnosis, gender, marital status, and race; the multivariate logistic regression model (Table 2) indicated that non-melanoma cancer survivors were 2.44 times (95% CI: 2.02, 2.95) more likely to believe that cancer had affected their overall health compared to melanoma survivors. In addition, compared to respondents treated with surgery only, survivors with combination therapy were 3.62 times (95% CI: 3.21, 4.08) were more likely to believe that cancer had affected their overall health. Survivors generally were less likely to believe that cancer had affected their overall health 10–20 years (OR = 0.57; 95% CI: 0.49, 0.66) after the time of diagnosis. Males were more likely (OR = 1.17; 95% CI: 1.05, 1.31) to believe that cancer had affected their overall health. Compared to married survivors, widowed responders were less likely (OR = 0.70; 95% CI: 1.05, 1.47) to report that their cancer had affected their health. Finally, as age increased, patients were less likely (OR: 0.99) to indicate that cancer had affected their overall health.

One hundred fifty-eight survivors (16.2%) of melanoma believed that having had cancer had affected their overall health compared with 2835 survivors (38.4%) of other cancers (Table 1). These melanoma survivors were more likely to have undergone combined treatments than surgery alone (29.9% vs. 6.1%); they were more frequently women (18.1% vs. 13.9%), and most of them had been diagnosed more recently than had survivors of other cancers (16.6 ± 9.0 vs. 19.3 ± 8.7 years).

There was no difference in effect on overall health relative to the interval since diagnosis among patients who had been treated with surgery alone (Table 3). However, for survivors who had undergone combined treatments, time since diagnosis appeared to be more important; 51.3% of those diagnosed within the previous decade said that cancer had affected their overall health, vs. 28.6% diagnosed within 10–20 years and 23.1% diagnosed more than 20 years earlier.

The melanoma survivors who had reported that cancer had affected their overall health did not differ from those who had reported that cancer had not affected their overall health with respect to sex, marital status, or ethnicity (Table 4). This was true both for those who had been treated with surgery alone and those who had undergone combined treatments.

At the time of the survey, 504 of the respondents (50.6%) were retired, and 327 (32.8%) reported they were still working. There were no major differences in working status with respect to sex, ethnicity, age at diagnosis, and time from diagnosis to survey. Of melanoma survivors who had undergone combined treatments, more women reported that cancer had affected their health than men; fewer were married in this category as well.

### Long-term health effects of cancer and its treatment

Survivors had been asked to report any health problems they had experienced since cancer treatment; the frequencies of perceived health problems for melanoma survivors treated with surgery alone or combined treatment and survivors of other cancers are outlined in Table 5.

The 577 melanoma survivors who had undergone surgery alone generally reported fewer health problems than did survivors of other cancers; the three most frequent symptoms reported were arthritis/osteoporosis (17.5%, significantly less than survivors of other cancers), cataracts (12.8%, similar to survivors of other cancers), and heart problems (10.6%, less than survivors of other cancers).

The 391 melanoma survivors who had undergone combined treatment were more likely to report lasting problems; the three most frequent symptoms reported were arthritis/osteoporosis (22.5%, similar to that of survivors of other cancers), cataracts (15.6%, similar to that of survivors of other cancers), and circulation problems (15.6%, less than survivors of other cancers). The frequency of self-reported heart problems in this group was similar to those for survivors of other cancers (12.5% vs. 14.5%).

Table 6 shows the influence of treatment on 10 most frequently reported long-term health problems in melanoma survivors who had been treated with surgery alone and combined treatment and the comparison of the frequency of these symptoms in the two groups. The absolute number of reported symptoms was small for most categories.

Survivors that were treated with combined treatment regimens complained of circulation problems, loss of memory, secondary cancers, skin problems, and abdominal pain significantly more frequently than survivors treated with surgery alone. Among the subgroup of melanoma survivors who reported that cancer had affected their health, survivors who received combined treatment complained of loss of memory and secondary cancers significantly more frequently than the survivors that were treated with surgery alone.

We evaluated whether some of the reported symptoms became more or less prominent over time among those who had undergone combined treatment (Table 7). Indeed, for the most part, there were no differences in perceived health problems during the first, second, or third decade after the diagnosis of melanoma. The only exceptions were cataracts and heart problems; whether this reflects an expected influence of older age remains to be determined.

Overall, 46.2% of survivors had reported that cancer had improved their family relationships, 4.6% had indicated that it had hurt their family relationships, and the remaining 49.2% had said it had had no effect on their family relationships (Table 8). The apparent effect of cancer on family relationships did not differ in terms of sex, ethnicity, age at diagnosis, age at survey, and time between survey and diagnosis. If the survivors believed that cancer had affected their overall health, they were more likely to report that cancer had hurt their family relationships. Those who had undergone combined treatments were also more likely to have reported that cancer had hurt their intimate relationships.

Individual chart review was carried out for the 243 melanoma survivors (132 women [54.3%] and 111 men [45.7%]) who had undergone combined treatment and who had provided identifying information. The records were reviewed for the types of treatment; the progress notes of follow-up appointments (including annual follow-up letters sent by the Department of Medical Informatics) were scrutinized for any documentation of long-term health effects. Metastatic disease was reported in 119 of the records (48.9%), melanoma recurrence in 47 (19.3%), and second primary cancers in 84 (34.5%). Table 9 outlines the common treatment modalities and the most frequent health problems reported by the survivors who had undergone those treatments. Heart problems were the most frequently reported; whether this represents a causal relationship rather than the effect of age on the frequency of heart disease could not be inferred.

In order to determine how health profiles of melanoma survivors compared with those of the general U.S. population, we compared their self-reported health conditions with age matched responses derived from the National Health Interview Study (NHIS) [11]. One caveat is that the NHIS population most likely includes some cancer survivors. Six health conditions in the NHIS matched to health conditions that were included in our survey: arthritis, cataracts, diabetes, hearing impairment, heart disease, and migraine headaches. In order to look at how age may have an effect on the health conditions we have classified the findings into three age groups based on the age at the time of survey. In addition, we have broken down the findings by type of treatment, combined treatment and surgery alone, in order to assess how treatment may have played a role in the survivors' lingering health effects. (Table 10)

Overall, melanoma survivors younger than 45 years at the time of the survey reported higher rates of arthritis (14.5%) and heart disease (10.8%) than age-matched responders in the NHIS (7.8% and 4.0%, respectively). Melanoma survivors between 45 and 64 years at the time of the survey indicated more kidney problems than their age-matched NHIS responders (3.7% vs. 1.5%). Survivors older than 65 years of age reported more health problems than their age-matched NHIS counterparts (33.6% vs. 26.2%).

After combined treatment, melanoma survivors reported more heart problems than their NHIS counterparts across all three age groups, 14.3% vs. 4.0% for responders younger than 45 years of age at the time of survey; 19.8% vs. 12.7% for the 45–64 years of age group; and 43.9% vs. 26.2% for the older group.

After surgery alone, melanoma survivors indicated more kidney problems than their age-matched NHIS counterparts across all three age groups, 5.7% vs. 0.6% for the younger group; 3.7% vs. 1.5% for the 45–64 years of age group, and 7.1% vs. 2.3% for the older group.

## Discussion

Our descriptive analysis indicates that, overall, melanoma survivors report relatively few lasting medical or social problems, even after often intensive combination treatment. However, age, gender, treatment, and several physiologic and psychological factors influence melanoma survivor's health profiles.

This study has several limitations. It is a subset of a larger cohort, and survey questions were not specific to melanoma survivors. In addition, the information includes self-reported problems that were only partly verifiable through medical record reviews, a limitation shared by the NHIS methodology. Nevertheless, the melanoma survivors answered the same questions as more than 10,000 survivors of other cancers, allowing for the opportunity to compare the health profiles of melanoma survivors with those of other cancer survivors, as well as NHIS responders.

Study of the long-term health outcomes of cancer survivors was conducted the earliest and most comprehensively in children [12-15]. Several investigators have noted that survivors of adult cancers experience significant lingering effects that influence their quality of life [16,17] and physiological health for many years [18]. Breast cancer survivors, for example, reported poorer functioning especially after chemotherapy [19]. In this group, prior chemotherapy was also identified as a risk factor for heart disease [20], the role of chest irradiation in the development of heart disease is less clear [21]. The symptoms and perceived problems often vary with the underlying malignancy [22]. Survivors of Hodgkin's disease have been found to have a higher rate of early mortality related to second primary cancers, heart disease, and infections than do those who have not had cancer [23]. Patients with testicular cancer fair well overall, both physiologically and psychologically, but they experience a number of lasting health problems [24-26]. In addition, researchers have characterized the late effects of chemotherapy and radiation on the pulmonary [27], circulatory [28], and cardiac [29,30] systems. Reports on melanoma are uncommon and limited to older feasibility studies [31] or case reports [32]. Close, life-long medical surveillance has been recommended [8] especially for patients with choroidal melanoma, who may have more quality of life problems [33,34].

In the present report, we used information obtained through a comprehensive survey of cancer survivors to determine the medical/physiologic and psychosocial characteristics and problems that affect long-term melanoma survivors.

In general, melanoma survivors reported fewer long-term health problems than did survivors of other cancers; this was particularly the case when they had been treated with surgery alone. In addition, they were less likely than survivors of other cancers to report that cancer had affected their overall health, although this response was found more frequently among those who had undergone combined treatment than surgery alone. Patients who believed cancer affected their overall health also believed cancer hurt their family and intimate relationships. As a group, melanoma survivors were younger than survivors of other cancers, and younger age at the time of diagnosis tends to correlate with increased distress from the cancer experience in survivors of other cancers, [2] but this was not obvious in this cohort. Systematic, prospective analyses of patients diagnosed with melanoma will be very important in order to define the long-term impact of this disease.

## Conclusion

Long-term melanoma survivors generally report that cancer had relatively little lasting effects on their health, although a number of disease-related and psychosocial factors influence their perception.

## Competing interests

The author(s) declare that they have no competing interests.

## Authors' contributions

CS had a major role in collecting and tabulating the data and in preparing the manuscript. TW and AL had an important role in carrying out the biostatistical analysis. MB had an important role in collecting and tabulating the data. RVS was the senior investigator with intimate participation in the design of data collection and analysis and in direction of manuscript preparation. All authors read and approved the final manuscript.

## Pre-publication history

The pre-publication history for this paper can be accessed here:



## Figures and Tables

**Table 1:** Comparison between survivors of melanoma and other cancers on selected demographic characteristics and effect on overall health

	Melanoma Survivors	Non-Melanoma Survivors
	n = 996	n = 7563

Gender		
Men	466 (46.8)	2888 (38.2)
Women	530 (53.2)	4675 (61.8)

Affected health		
Yes	158 (16.2)	2835 (38.4)
No	820 (83.4)	4546 (61.6)

	Mean ± SD	Mean ± SD

Age @ Diagnosis	44.8 ± 12.8 yrs	49.4 ± 14.2 yrs
Age @ Survey	63.7 ± 12.8 yrs	64.7 ± 13.4 yrs
Time from diagnosis	18.8 ± 8.8 yrs	15.2 ± 8.9 yrs

SD = standard deviation

**Table 2:** Multivariate logistic model for health affected versus non-health affected

Effect		OR	OR 95% Confidence Limits		P-value
Type of Cancer (ref: Melanoma)					
	Non-Melanoma	2.44	2.02	2.95	<.0001
Treatment Group (ref: Surgery only)					
	Combined Therapy	3.62	3.21	4.08	<.0001
Years from Diagnosis (ref: <10 years)					
	10 – 20 yrs	0.65	0.58	0.73	0.0048
	> 20 yrs	0.57	0.49	0.66	<.0001
Gender (ref: Female)					
	Male	1.17	1.05	1.31	0.0045
Marital (ref: Married)					
	Widow	0.70	0.59	0.82	<.0001
	Divorced	1.25	1.05	1.47	0.0007
	Never married	1.06	0.80	1.40	0.4738
Race (ref: Other)					
	White	0.98	0.81	1.18	0.7941
Age @ diagnosis		0.989	0.99	0.99	<.0001

OR = Odds Ratio

Ref = reference group

**Table 3:** Influence of time since melanoma diagnosis and age at diagnosis on overall health by treatment

Combined Treatment			
	Total n = 384	Health Affected n = 117	Health Not affected n = 267

	n (%)	n (%)	n (%)

Time from diagnosis			
< 10 years	78 (19.9)	39 (51.3)	37 (48.7)
10–20 years	128 (32.7)	36 (28.6)	90 (71.4)
> 20 years	185 (47.3)	42 (23.1)	140 (76.9)
			
		Mean ± SD	Mean ± SD
Age at diagnosis (years)		44.7 ± 14.0	45.6 ± 12.1
Time from diagnosis (years)		16.6 ± 9.2	20.1 ± 8.4

Surgery Alone			

	Total n = 566	Health Affected n = 35	Health Not Affected n = 531

	n (%)	n (%)	n (%)

Time from diagnosis			
< 10 years	96 (16.6)	7 (7.4)	87 (92.6)
10–20 years	249 (43.2)	17 (7.0)	227 (93.0)
> 20 years	232 (40.2)	11 (4.8)	217 (95.2)
			
		Mean ± SD	Mean ± SD
Age at diagnosis (years)		42.4 ± 12.5	44.4 ± 12.3
Time from diagnosis (years)		16.8 ± 8.4	18.7 ± 8.7

SD = Standard deviation

**Table 4:** Influence of demographics on overall health by treatment

**Combined Treatments**		
	Health Affected n = 117	Health Not Affected n = 267

Demographics	n (%)	n (%)

Sex		
Male	54 (29.7)	128 (70.3)
Female	63 (31.2)	139 (68.8)
Marital status		
Married	89 (31.8)	191 (68.2)
Widowed	15 (24.2)	47 (75.8)
Divorced	6 (19.4)	25 (80.7)
Never married	7 (70.0)	3 (30.0)
Ethnicity		
White	117 (31.0)	260 (69.0)
Nonwhite	0 (0.0)	7 (100.0)

**Surgery Alone**		

	Health Affected n = 35	Health Not Affected n = 531

Demographics	n (%)	n (%)

Sex		
Male	8 (3.0)	257 (97.0)
Female	27 (9.0)	274 (91.0)
Marital status		
Married	24 (5.6)	404 (94.4)
Widowed	4 (5.3)	72 (94.7)
Divorced	7 (15.6)	38 (84.4)
Never married	0 (0.0)	17 (100.0)
Ethnicity		
White	34 (6.1)	531 (93.9)
Nonwhite	1 (8.3)	11 (91.7)

**Table 5:** Comparison of 10 most reported specific health problems in melanoma survivors and survivors of all other cancers

Health Problems	Melanoma Survivors		Survivors of all other cancers
	Surgery Alone n = 577	Combined Treatments n = 391	n = 7563
		
Arthritis/Osteoporosis	101 (17.5%)	88 (22.5%)	2012 (26.6%)
Hearing Problems	50 (8.7%)	54 (13.8%)	892 (11.8%)
Cataracts	74 (12.8%)	61 (15.6%)	1111 (14.7%)
Circulation Problems	42 (7.3%)	61 (15.6%)	803 (10.6%)
Heart problems	61 (10.6%)	46 (12.5%)	1093 (14.5%)
Skin problems	34 (5.9%)	43 (11.0%)	415 (5.5%)
Loss of Memory	8 (1.4%)	42 (10.7%)	767 (10.1%)
Thyroid Problems	29 (5.0%)	29 (7.4%)	724 (9.6%)
Kidney Problems	32 (5.6%)	29 (7.4%)	885 (11.7%)
Abdominal Pain	14 (2.4%)	23 (5.9%)	532 (7.0%)

**Table 6:** Health problems of melanoma survivors: influence of treatment

	All Melanoma Survivors		Health Affected by Cancer	
	Surgery Alone	Combined Treatments	Surgery Alone	Combined Treatments

	n = 577	n = 391	n = 35	n = 117
	n (%)	n (%)	n (%)	n (%)
Arthritis/Osteoarthritis	101 (17.5%)	88 (22.5%)	8 (22.8%)	36 (30.7%)
Other/Subsequent Cancers	36 (6.2%)	76 (19.4%)	2 (5.7%)	30 (25.6%)
Cataracts	74 (12.8%)	61 (15.6%)	3 (8.5%)	14 (11.9%)
Circulation Problems	42 (7.3%)	61 (15.6%)	8 (22.8%)	30 (25.6%)
Hearing Problems	50 (8.6%)	54 (13.8%)	3 (8.5%)	17 (14.5%)
Loss of Memory	8 (1.4%)	42 (10.7%)	1 (2.8%)	23 (19.6%)
Skin Problems	34 (5.8%)	43 (10.9%)	2 (5.7%)	20 (17.1%)
Thyroid Problems	29 (5.1%)	29 (7.4%)	5 (14.3%)	12 (10.3%)
Abdominal Pain	14 (2.4%)	23 (5.8%)	3 (8.5%)	15 (12.8%)
Heart Problems	61 (10.6%)	46 (11.7%)	1 (2.8%)	13 (11.1%)

**Table 7:** Association between time since diagnosis and health problems for melanoma survivors who had undergone combined treatments

	Time Between Diagnosis and Survey		
	
	< 10 yrs n = 78	10–20 yrs n = 128	> 20 yrs n = 185
Health Problems	n (%)	n (%)	n (%)

Arthritis/osteoarthritis	15 (19.2)	29 (22.6)	44 (23.7)
Hearing problems	8 (10.3)	20 (15.6)	26 (14.1)
Cataracts	5 (6.4)	17 (13.3)	39 (21.1)
Circulation problems	11 (14.1)	21 (16.4)	29 (15.6)
Heart problems	3 (3.8)	18 (14.1)	28 (15.1)
Skin problems	12 (15.4)	13 (10.2)	18 (9.7)
Loss of memory	13 (16.6)	12 (9.4)	17 (9.2)
Thyroid	6 (7.8)	11 (8.6)	12 (6.5)
Kidney problems	6 (7.8)	6 (4.7)	17 (9.2)
Abdominal pain	7 (8.9)	7 (5.5)	9 (4.8)

**Table 8:** Family relationships of melanoma survivors

	Health Affected	Health Not Affected
	n (%)	n (%)

Combined:		
Treatment:		
Improved Family Relationships	61 (31.6)	132 (68.4)
No Improvement	50 (31.1)	111 (68.9)

Hurt Family Relationships	18 (66.7)	9 (33.3)
No Harm	94 (27.6)	246 (72.4)

Improved Intimate Relationships	29 (32.2)	61 (67.8)
No Improvement	78 (31.8)	167 (68.2)

Hurt Intimate Relationships	38 (65.5)	20 (34.5)
No Harm	70 (24.1)	220 (75.9)

Surgery Alone:		
Improved Family Relationships	11 (4.5)	231 (95.5)
No Improvement	24 (8.4)	260 (91.6)

Hurt Family Relationships	8 (42.1)	11 (57.9)
No Harm	24 (4.5)	504 (95.5)

Improved Intimate Relationships	5 (4.2)	113 (95.8)
No Improvement	29 (7.5)	356 (92.5)

Hurt Intimate Relationships	9 (36.0)	19 (64.0)
No Harm	22 (4.5)	465 (95.5)

**Table 9:** Relationship between treatments and health problems

Treatment	Number of Patients*	Most Frequently Reported Health Effects		
Dacarbazine	89	Cardiovascular (12%),	lymphedema (8%),	arthritis (4%)
Phenylalanine mustard	65	Cardiovascular (10%),	lymphedema (11%),	arthritis (5%)
Radiation therapy	63	Radiation damage (8%),	cardiovascular (8%),	diabetes (5%)
Interferon	52	Cardiovascular (6%),	skin problems (6%),	lymphedema (4%)
Bacillus Calmette-Guerin	42	Cardiovascular (14%),	lymphedema (12%),	ulcer problems (5%)
Cisplatin	31	Arthritis (6%),	cardiovascular (6%),	neuropathy (6%)
Actinomycin-D	22	Cardiovascular (36%),	lymphedema (14%),	arthritis (9%)
Vinblastine	21	Neuropathy (9%),	arthritis (5%),	
Interleukin	18	Arthritis (6%),	cardiovascular (6%),	
Vincristine	17	Cardiovascular (18%),		

* A few patients received more than one agent.

**Table 10:** Health status of melanoma survivors versus that of NHIS participants

**A. Overall**						
	< 45 Survivors* n = 83	< 45 NHIS^1 ^n = 108114	45–64 Survivors* n = 405	45–64 NHIS^1 ^n = 64650	≥ 65 Survivors* n = 508	≥ 65 NHIS^1 ^n = 17809

Arthritis	14.4%	7.8%	16.1%	28.8%	23.2%	44.6%
Diabetes Mellitus	2.4%	1.9%	3.5%	9.3%	10.0%	17.1%
Hearing Loss	2.4%	6.7%	3.7%	17.5%	18.1%	29.7%
Heart Disease	10.8%	4.0%	16.1%	12.7%	33.6%	26.2%
Kidney Problems	3.6%	0.6%	3.7%	1.5%	8.5%	2.3%
Liver Problems	2.4%	0.8%	1.2%	1.9%	0.9%	1.3%
Migraine Headaches	3.6%	17.6%	3.5%	15.3%	0.7%	7.8%

**B. Combined Treatments**						

	< 45 Survivors* n = 28	< 45 NHIS^1 ^n = 108114	45–64 Survivors* n = 156	45–64 NHIS^1 ^n = 64650	≥ 65 Survivors* n = 207	≥ 65 NHIS^1 ^n = 17809
Arthritis	21.4%	7.8%	19.2%	28.8%	25.1%	44.6%
Diabetes Mellitus	3.7%	1.9%	2.5%	9.3%	10.6%	17.1%
Hearing Loss	7.2%	6.7%	4.5%	17.5%	21.7%	29.7%
Heart Disease	14.3%	4.0%	19.8%	12.7%	43.9%	26.2%
Kidney Problems	0.0%	0.6%	3.8%	1.5%	11.1%	2.3%
Liver Problems	0.0%	0.8%	2.5%	1.9%	2.4%	1.3%
Migraine Headaches	7.2%	17.6%	4.5%	15.3%	0.5%	7.8%

**C. Surgery Alone**						

	< 45 Survivors* n = 53	< 45 NHIS^1 ^n = 108114	45–64 Survivors* n = 240	45–64 NHIS^1 ^n = 64650	≥ 65 Survivors* n = 284	≥ 65 NHIS^1 ^n = 17809
Arthritis	11.3%	7.8%	13.7%	28.8%	21.8%	44.6%
Diabetes Mellitus	1.9%	1.9%	4.2%	9.3%	9.8%	17.1%
Hearing Loss	0.0%	6.7%	2.9%	17.5%	15.1%	29.7%
Heart Disease	9.4%	4.0%	13.3%	12.7%	27.1%	26.2%
Kidney Problems	5.7%	0.6%	3.7%	1.5%	7.1%	2.3%
Liver Problems	3.8%	0.8%	0.4%	1.9%	0.0%	1.3%
Migraine Headaches	1.9%	17.6%	2.9%	15.3%	1.1%	7.8%

* At time of survey

^1 ^**NHIS data from Vital and Health Statistics Series 10, Number 222**. National Health Interview Survey, US Department of Health and Human Services, Hyattsville MD. 2002

## References

[B1] (2005). Facts about cancer survivorship. National Cancer Institute Office of Cancer Survivorship.

[B2] Lotze MT, Dallal RM, DeVita VT, Rosenberg SA, Hellman S (2001). Cutaneous melanoma. Principles and practices of oncology.

[B3] (2005). What are the key statistics about Melanoma?. American Cancer Society.

[B4] Meric JB, Rixe O, Khayat D (2003). Metastatic malignant melanoma. Drugs Today (Barc).

[B5] (2005). Melanoma Fact Sheet. The Melanoma Research Foundation.

[B6] Balch CM, Reintgen DS, Kirkwood JM, Houghton A, Peters L, Ang KK, DeVita VT, Rosenberg SA, Hellman S (1997). Cutaneous melanoma. Principles and practices of oncology.

[B7] Berwick M, Weinstock MA, St Louis (2003). Epidemiology current trends in cutaneous melanoma.

[B8] Christianson DF, Anderson CM (2003). Close monitoring and lifetime follow up is optimal for patients with a history of melanoma. Semin Oncol.

[B9] Hewitt M, Greenfield S, Stovall E (2006). From Cancer Patient to Cancer Survivor: Lost in Transition.

[B10] Schultz PN, Stava C, Beck M, Vassilopoulou-Sellin R (2003). Health profiles in 5836 long-term cancer survivors. Int J Cancer.

[B11] (2002). NHIS data from Vital and Health Statistics Series 10, Number 222. National Health Interview Survey, US Department of Health and Human Services, Hyattsville MD.

[B12] Meadows AT, Hobbie WL (1986). The medical consequences of care. Cancer.

[B13] Reid, Zietz H, Jaffe N (1995). Late effects of cancer treatment in children. Pediatr Dent.

[B14] Marina N (1997). Long-term survivors of childhood cancer. The medical consequences of cure. Pediatr Clin North Am.

[B15] Stevens MC, Mahler H, Parks S (1998). The health status of adult survivors of cancer in childhood. Eur J Cancer.

[B16] Gotay CC, Muraoka MY (1998). Quality of life in long-term survivors of adult-onset cancers. J Natl Cancer Inst.

[B17] Welch-McCaffrey D, Hoffman B, Leigh SA, Loescher LJ, Meyskens FL (1998). Surviving adult cancers. Part 2: psychosocial implications. Ann Intern Med.

[B18] Loescher LJ, Welch-McCaffrey D, Leigh SA, Hoffman B, Meyskens FL (1998). Surviving adult cancers. Part 1 physiologic effects. Ann Intern Med.

[B19] Ganz PA, Desmond KA, Leedham B, Rowland JH, Meyerowitz BE, Belin TR (2002). Quality of life in long-term, disease-free survivors of breast cancer: a follow-up study. J Natl Cancer Inst.

[B20] Bonneterre J, Roche H, Kerbrat P, Fumoleau P, Goudier MJ, Fargeot P, Montcuquet P, Clavere P, Barats JC, Monnier A, Veyret C, Patchary J, Van Praagh I, Chapelle-Marcillac I (2004). Long-term cardiac follow-up in relapse-free patients after six courses of fluorouracil, epirubicin, and cyclophosphamide, with either 50 or 100 mg of epirubicin, as adjuvant therapy for node-positive breast cancer: French Adjuvant Study Group. J Clin Oncol.

[B21] Rutqvist LE, Liedberg A, Hammer N, Dalberg K (1998). Myocardial infarction among women with early-stage breast cancer treated with conservative surgery and breast irradiation. Int J Radiat Oncol Biol Phys.

[B22] Bloom JR, Fobair P, Gritz E, Wellisch D, Speigel D, Varghese A, Hoppe R (1993). Psychosocial outcomes of cancer: a comparative analysis of Hodgkin's disease and testicular cancer. J Clin Oncol.

[B23] Hudson MM, Poquette CA, Lee J, Greenwald CA, Shah A, Luo X, Thompson EI, Wilimas JA, Kun LE, Crist WM (1998). Increased mortality after successful treatment for Hodgkin's disease. J Clin Oncol.

[B24] Vaughn DJ, Gignac GA, Meadows AT (2002). Long-term medical care of testicular cancer survivors. Ann Intern Med.

[B25] Joly F, Heron JF, Kalusinski L, Bottet P, Brune D, Allouache N, Mace-Lese'h J, Couette JE, Peny J, Henry-Amar M (2002). Quality of life in long-term survivors of testicular cancer: a population-based case-control study. J Clin Oncol.

[B26] Fossa SD, Dahl AA, Loge JH (2003). Fatigue, anxiety, and depression in long-term survivors of testicular cancer. J Clin Oncol.

[B27] Theuws JCM, Muller SH, Seppenwoolde Y, Kwa SL, Boersma LJ, Hart GA, Baas P, Lebesque JV (1999). Effect of radiotherapy and chemotherapy on pulmonary function after treatment for breast cancer and lymphoma: a follow-up study. J Clin Oncol.

[B28] Dorresteijn LDA, Kappelle AC, Boogerd W, Klokman WJ, Balm AJ, Keus RB, Van Leeuwen FE, Bartelink H (2002). Increased risk of ischemic stroke after radiotherapy on the neck in patients younger than 60 years. J Clin Oncol.

[B29] Shapiro CL, Hardenbergh PH, Gelman R, Blanks D, Hauptman P, Recht A, Hayes DF, Harris J, Henderson IC (1998). Cardiac effects of adjuvant doxorubicin and radiation therapy in breast cancer patients. J Clin Oncol.

[B30] Trivedi A, Hannan MA (2004). Radiation and cardiovascular diseases. J Environ Pathol Toxicol Oncol.

[B31] Sigurdardottir V, Bolund C, Brandberg Y, Sullivan M (1993). The impact of generalized malignant melanoma on quality of life evaluated by the EORTC questionnaire technique. Quality of Life Research.

[B32] Gruss C, Geissler A, Schalke B, Landthaler M, Stolz W (2002). Severe neurological disabilities after complete remission of advanced malignant melanoma following fotemustine therapy in combination with total brain irradiation. Melanoma Res.

[B33] Reimer J, Esser J, Fleiss A, Hessel A, Anastassiou G, Krausz M, Bornfeld N, Franke GH (2003). Quality of life in patients with malignant choroidal melanoma after radiotherapy. Graefes Arch Clin Exp Opthalmol.

[B34] Brown JE, King MT, Butow PN, Dunn SM, Coates AS (2000). Patterns over time in quality of life, coping, and psychological adjustment in late stage melanoma patients: an application of multilevel models. Qual Life Res.

